# Why pyridoxal phosphate could be a functional predecessor of thiamine pyrophosphate and speculations on a primordial metabolism†

**DOI:** 10.1039/d4cb00016a

**Published:** 2024-04-18

**Authors:** Andreas Kirschning

**Affiliations:** a Institute of Organic Chemistry, Leibniz University Hannover, Schneiderberg 1B 30167 Hannover Germany andreas.kirschning@oci.uni-hannover.de; b Uppsala Biomedical Center (BMC), University Uppsala, Husargatan 3 752 37 Uppsala Sweden

## Abstract

The account attempts to substantiate the hypothesis that, from an evolutionary perspective, the coenzyme couple pyridoxal phosphate and pyridoxamine phosphate preceded the coenzyme thiamine pyrophosphate and acted as its less efficient chemical analogue in some form of early metabolism. The analysis combines mechanism-based chemical reactivity with biosynthetic arguments and provides evidence that vestiges of “TPP-like reactivity” are still found for PLP today. From these thoughts, conclusions can be drawn about the key elements of a primordial form of metabolism, which includes the citric acid cycle, amino acid biosynthesis and the pentose phosphate pathway.

## Abstract 2

On the basis of mechanistic and biosynthetic considerations, arguments are put forward as to why, from an evolutionary point of view, the coenzyme pyridoxal phosphate preceded thiamine pyrophosphate.

## Graphical abstract

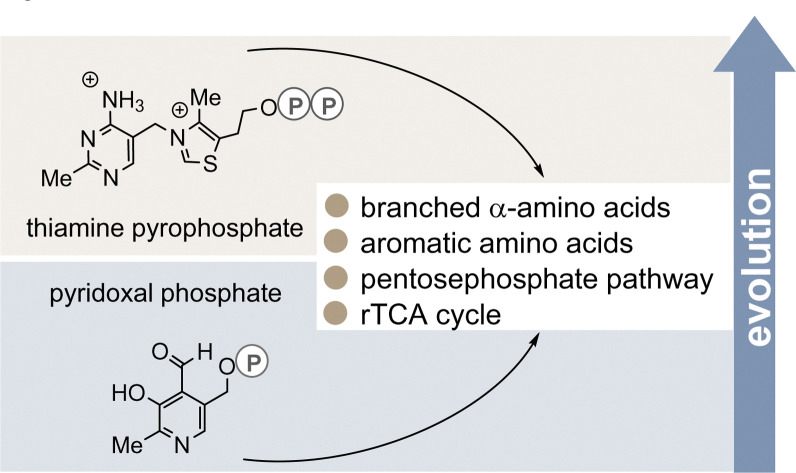

## 1. Introduction

Discussions on primordial metabolic processes that may have functioned in primitive life forms before the emergence of the last unified common ancestor (LUCA) are highly speculative. Ideas about the transitional state between prebiotic chemistry and the first forms of heterotrophic systems of life can be captured by different approaches. One of these is to use the evolution of coenzymes and cofactors as a guideline. As early as 1976, White III proposed that coenzymes are the surviving vestiges of nucleic acid enzymes due to the fact that many coenzymes contain structural elements derived from RNA nucleotides.^1^ He claimed that their occurrence preceded the evolution of ribosomal protein synthesis. However, this aspect has never been a major issue or driving force in hypothesizing the origin of life. This neglect has just recently begun to change to a certain degree.^2^ In fact, coenzymes and cofactors must be very ancient because (a) they occur in all kingdoms of life, (b) a recently substantiated model of LUCA by phylogenetic and bioinformatics analyses^3^ must have used the majority of coenzymes and cofactors known today and (c) the biosynthesis of proteinogenic amino acids and the enzymatic formation of coenzymes represent a classic case of a “chicken and egg” problem.^4^ From a chemical point of view, they can be considered the most chemical species that nature has developed, since their almost sole purpose is to promote chemical reactions until today.

If this chemical role is of such universal importance for life, then the question arises whether a biosynthetic access was at hand for all coenzymes and cofactors at the same time. There are different ideas about this topic – one approaches it by analyzing the biosynthetic pathways of individual coenzymes and cofactors with respect to the requirements of other coenzymes.^5^

**Fig. 1:**
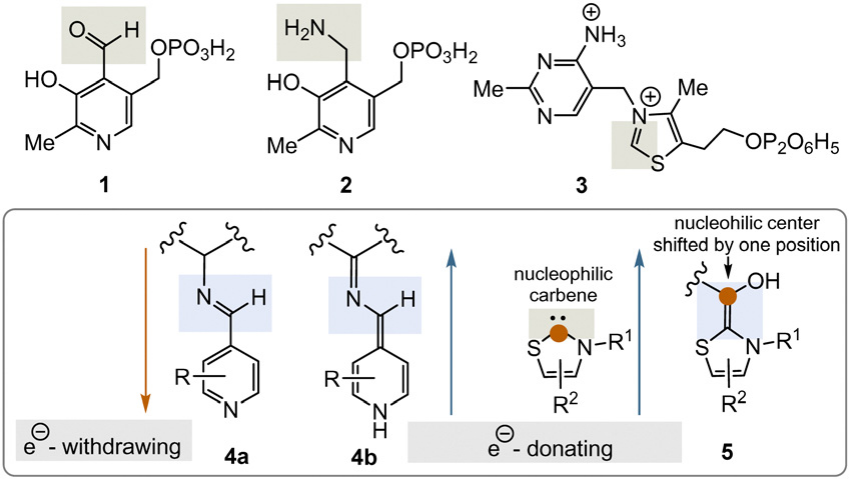
Structures of the PLP/PMP pair 1/2 and TPP 3 as well as the biosynthetic intermediates 4a, b and 5 that explain their general reactivity patterns (phosphate groups are presented in a fully protonated form or simply as an encircled P throughout the text).

Two important coenzymes, pyridoxal phosphate (PLP, 1), its “twin” pyridoxamine phosphate (PMP, 2), and thiamine pyrophosphate (TPP, 3), play a central role in primary metabolism (specifically amino acids, carbohydrates, and the tricarboxylate acid cycle (TCA cycle)) as well as in the elaboration of secondary metabolites. In this short review, chemical and biosynthetic considerations are used to argue that PLP appeared before TPP during the evolution of life and that, chemically speaking, PLP might have fulfilled the chemical role of TPP before the arrival of TPP. The chemical part of this reasoning will focus primarily on the reactive intermediates 4a, b and 5 or analogues (Fig. 1), while elements of the primary metabolism will provide biosynthetic arguments.

It is the author's intention to provide new ideas and thoughts on how a primordial metabolism of simple living systems might have looked like before LUCA by analyzing the evolution of the two coenzymes mentioned here. Any primordial metabolism hypothesis, including those based on phylogenetic analyses, is fraught with the problem of speculation and prone to controversy.

## 2. The coenzymes pyridoxal phosphate and thiamine pyrophosphate

**Scheme 1:**
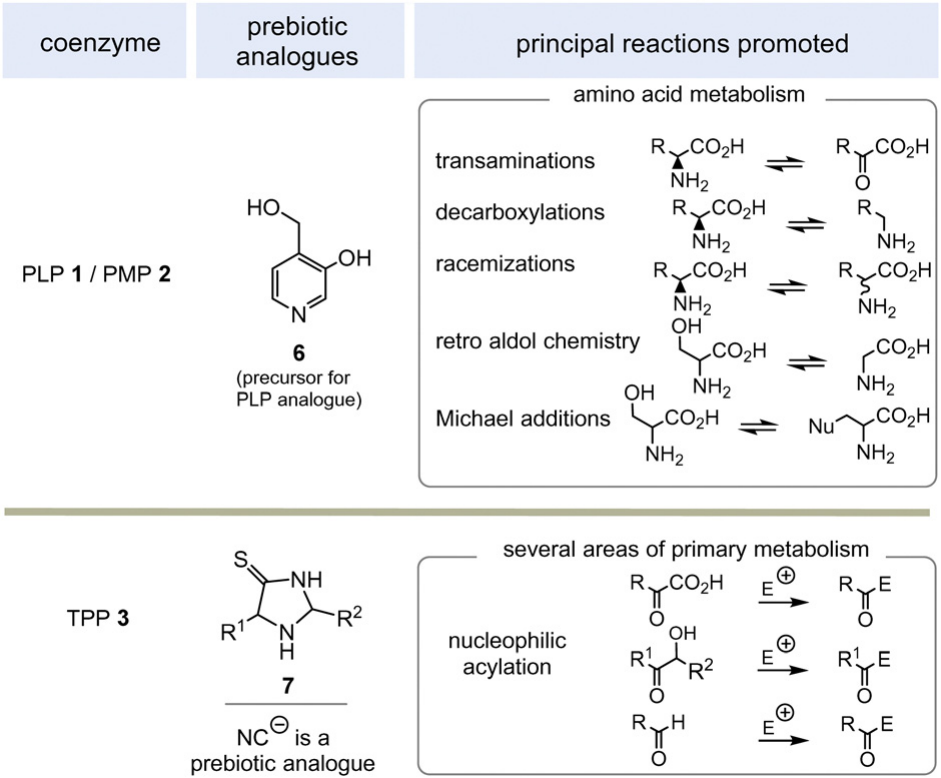
Prebiotically synthesised simplified analogue 6 and chemistries they promote in life (E = electrophile such as H^+^, SCoA esters, aldehydes and others).

PLP 1 and PMP 2 play a predominant role in amino acid metabolism, while TPP 3 acts as an acyl anion carrier in various sections of primary metabolism, particularly carbohydrate and amino acid metabolism. In pyridoxal phosphate 1, the pyridine ring serves as an electron acceptor for the aldehyde group or for the imine (structure 4a) formed by condensation with α-amino acids. Having accepted electrons, the situation changes and the dihydropyridine moiety present in structure 4b becomes an electron donor.^6^ These electronic features allow promotion of a series of chemical transformations such as transaminations,^7*a*^ decarboxylations with the formation of biogenic amines, and racemisations as an entry into the world of D-amino acids, but also retro-aldol reactions and Michael additions are promoted by this pair of coenzymes (Scheme 1).^7^

TPP 3 uses two main types of substrates to generate acyl anion synthons, aldehydes and α-ketocarboxylic acids. Specifically, TPP is involved in the formation of acetyl-CoA from pyruvate, which serves as an entry point into the TCA cycle. It also plays a role within the TCA cycle and its putative evolutionary precursor, the reductive (or reverse) TCA (rTCA) cycle. In addition, it is required for the biosynthesis of the branched-chain α-amino acids l-valine, l-leucine, and l-isoleucine, as well as the aromatic amino acids l-phenylalanine, l-tyrosine and l-tryptophan; the latter amino acids are formed *via* the shikimic acid pathway.^8^ TPP 3 plays another prime role in carbohydrate biosyntheses where it also acts as a promotor of acyl anion chemistry in transketolase-mediated transformations. In essence, TPP promotes the “Umpolung” of aldehydes and ketones and mechanistically the “Breslow” intermediate 5 has to be given a central role.

## 3. Arguments

### 3.1 Argument 1: a “chicken-and-egg” problem and LUCA does not solve it

What was first? Coenzymes like PLP and TPP or proteins?^9^ The biosynthesis of all twenty proteinogenic α-amino acids requires at least one, often multiple, coenzyme(s), and PLP is almost always involved, whereby the starting building blocks are predominantly recruited from the TCA cycle. However, all coenzymes and cofactors are the result of enzyme-mediated biosyntheses, making the protein–coenzyme pair a case of causal circularity or a “chicken and egg” problem. According to these considerations, not only amino acids but also coenzymes must have been present early in the origin and evolution of life. Can our current view on LUCA give us a clue to solve this problem of causal circularity? Martin's model of LUCA^3^ does include the biosyntheses and thus the supply of PLP and TPP. If PLP and TPP already existed at this early date, then several fundamental questions arise. When did these coenzymes appear on the scene? Did simpler predecessors exist even earlier and did the biosyntheses for PLP and TPP emerge at the same time?

Regarding question 1, White III already provided an answer,^1^ especially if a fundamental theory about the origin of life, the “RNA first” theory, is correct.^10^ In fact, a number of coenzymes can be seen as a link between the “RNA first” and “metabolism first” theories, as they contain structural elements of RNA but act as mediators and catalysts in metabolism. When RNA is combined with coenzymes or cofactors, the catalytic potential of RNA can theoretically be expanded. Indeed, complexes composed of RNA and coenzymes or cofactors are known and are called riboswitches. However, their biological role today is a regulatory and not a catalytic one.^2^ The second question relates the hypothetical transformation from a prebiotic chemical world to the earliest forms of life that existed well before the emergence of LUCA. For pyridoxal phosphate 1, the chemical synthesis of the simplified analogue 6 was reported.^11^ Trapp and coworkers searched for prebiotically plausible routes to heterocyclic carbenes that might have served as primordial TPP-like organocatalysts. Imidazolidine-4-thiones form from mixtures of aldehydes or ketones in the presence of ammonia, cyanide and H_2_S and allow α-alkylation of aldehydes.^12*a*^ Interestingly, under dynamic system chemistry-like conditions, they are able to adapt to their environment by “mutation” of their own structure with an effect on their catalytic properties.^12*b*^ Additionally, the cyanide anion can be considered to be the prebiotically simplest TPP alike species as it is also able to promote acyl anion chemistry (*e.g.* the benzoin reaction).^12*c*^

### 3.2. Argument 2: on the biosynthesis of pyridoxal phosphate and thiamine pyrophosphate – PLP was first!

**Scheme 2:**
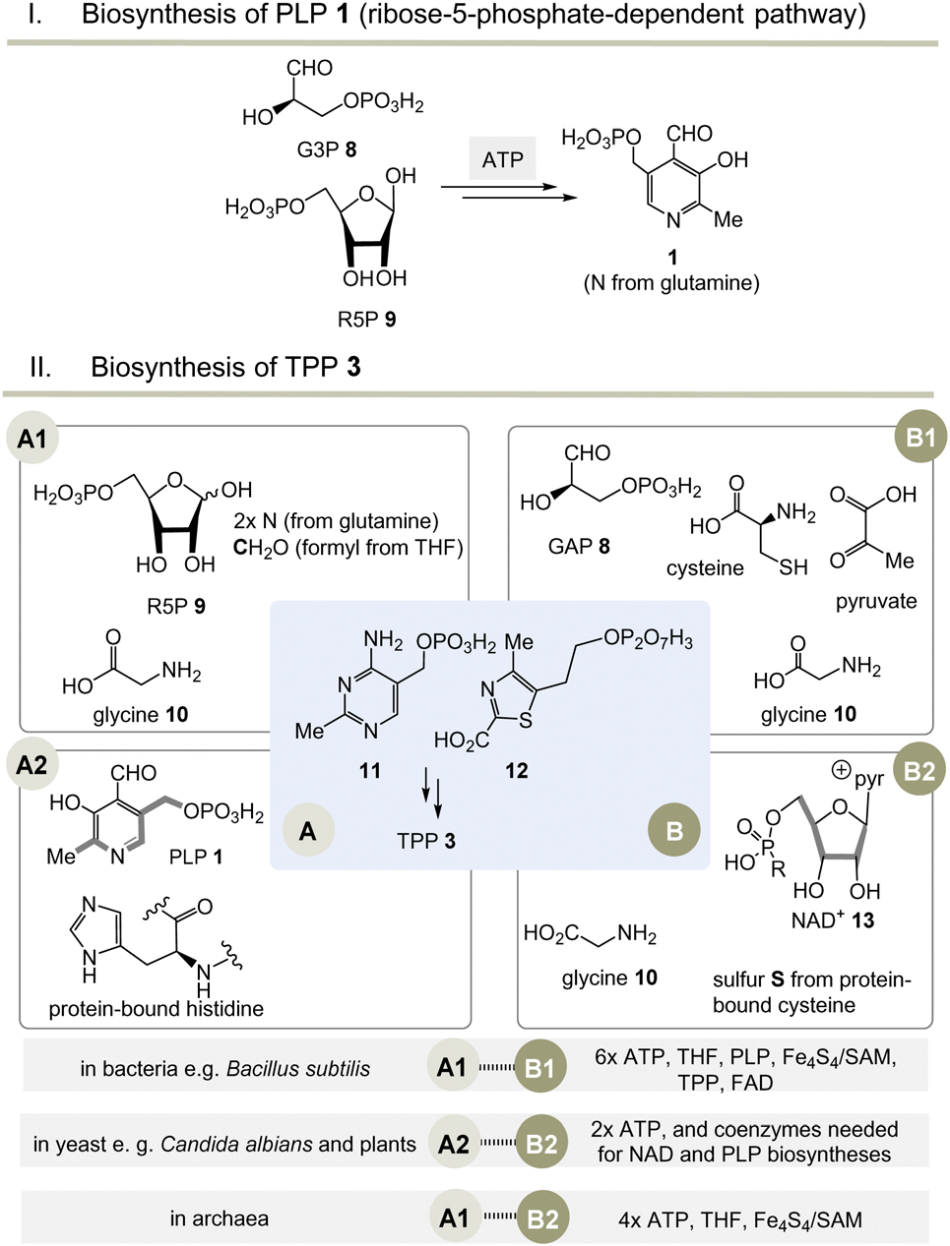
(I) Biosynthesis of PLP 1 (only the R5P pathway is depicted); (II) three biosynthetic variants to the two key intermediates 11 and 12 (A1–B1, A2–B2 and A1–B2) and hence to TPP 3 (for details see ref. 5). ^a ^Coenzymes: ATP = adenosine triphosphate, SAM = *S*-adenosylmethionine, FAD = flavine adenosine dinucleotide, THF = tetrahydrofolic acid, NAD^+^ = nicotinamide dinucleotide. Cofactor: Fe_4_S_4_ = iron–sulfur cluster.

Moving to the field of biosynthesis, one has to acknowledge that two biosynthetic pathways for PLP have been identified (Scheme 2I).^13^ The ribose-5-phosphate-dependent (or deoxy-xylulose-phosphate-independent) pathway, that is found, for example, in *Bacillus subtilis* but has also been detected in archaea, fungi and plants, uses glyceraldehyde-3-phosphate (G3P, 8) and ribose-5-phosphate (R5P, 9 in equilibrium with ribulose-5-phosphate (Ru5P)) as carbon sources and the nitrogen atom is provided by glutamine.^14^ The entire metabolic pathway relies only on ATP to regenerate glutamine from glutamate, while other coenzymes are not involved in its biosynthesis.

The second metabolic pathway studied in *E. coli* starts from 3-hydroxy-aminoacetone phosphate and 1-deoxy-xylulose 5-phosphate. This pathway requires a series of coenzymes for individual enzymatic steps, PLP included, so that this route can be called a “straggler” pathway. Therefore, it is of no relevance when discussing issues related to the evolution of protocells.

Nature has evolved three different biosynthetic variants to TPP 3,^15^ all of which are terminated by a substitution reaction between hydroxymethyl-pyrimidine phosphate (HMP-P, 11) and hydroxyethylthiazole phosphate (HET-P, 12) followed by phosphorylation (Scheme 2, II).^16^ These two fragments are biosynthesised by two independent routes. Either ribose 5-phosphate (R5P, 9) and glycine 10 (fragment route A1) or alternatively protein-bound histidine and PLP 1 can lead to HMP-P 11 (fragment route A2). The second fragment HET-P 12 is formed either from glycine 10, cysteine, pyruvate and G3P 8 (fragment route B1) or alternatively from glycine 10 and five carbon atoms of ribose that come from the coenzyme NAD^+^13 (fragment route B2).^17^

The selection and role of the coenzymes required for each biosynthetic pathway to both fragments 11 and 12 allows an assessment of the relative evolutionary appearance of the three pathways. The bacterial fragment biosynthesis route B1 to 11 requires TPP for its own generation (pyruvate + G3P 8 yields 1-deoxy-xylulose-5-phosphate), making it an evolutionary irrelevant route.^18^

Unusually, the biosynthetic subroutes A2 and B2 use the coenzymes PLP and NAD^+^13 as building blocks, implying that these two subroutes to TPP are evolutionarily younger than those of PLP/PMP 1/2 or nicotinamide dinucleotide 13. As a consequence of this analysis, it can be concluded that the biosynthesis of PLP appeared on the stage of life long before those that provide TPP. Thus, TPP can be regarded a “latecomer” among the group of coenzymes.^5^

### 3.3. Argument 3: how PLP might have served as a predecessor of TPP – a mechanistic view

The considerations outlined above and the fact that TPP 3 is assigned a central role in a number of fundamental areas of primary metabolism imply that it makes sense to explore chemical alternatives to TPP. Nature provides a clue as it was found that the bacteria *Borrelia* and *Rickettsia* do not require TPP 3 for their metabolism,^19^ challenging the paradigm that this vitamin is essential for life. However, this fact should only be taken as an indicator, simply because from an evolutionary point of view the two organisms could have lost the TPP-related metabolism as a result of their adaptations.

**Scheme 3:**
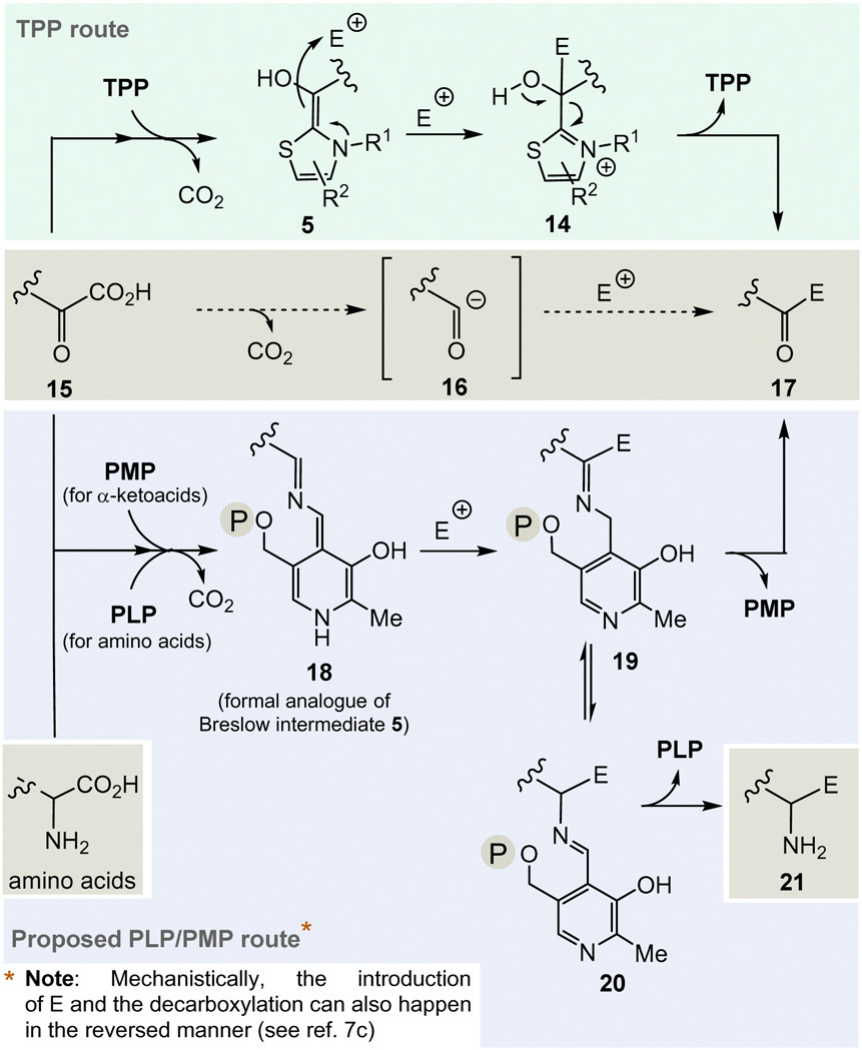
Top: Mechanistic route from 15 to 17*via* the Breslow intermediate 6 that is catalyzed by TPP 3. Central: Formal acyl “Umpolung“ after decarboxylation of α-ketocarboxylates 15 and formation of ketones 17. Bottom: Proposed mechanistic route from 15 to 17 or from α-amino acids that likewise could be promoted by PLP/PMP 1/2 and side route to amines 21*via*20 by including a transamination step.

And it is here that the author proposes PLP/PMP 1/2 as a reasonable chemical substitute. Mechanistically, however, the generation of an analogue of the TPP-dependent “Breslow” intermediate 5^20^ must be formulated for PLP/PMP 1/2 (Scheme 3).

Noteworthily, the transamination reaction permits choosing either amino acids or α-ketocarboxylates 15 as starting points. The most obvious evidence, that the PLP/PMP pair can actually act as a formal acyl anion equivalent, is decarboxylation of α-amino acids (*e.g.* when starting with α-ketocarboxylates) where the proton can be regarded to be the electrophile (E^+^).^7^ Decarboxylation of the PMP adduct yields imine 18. This intermediate can be regarded to be a formal equivalent of the Breslow intermediate 5. If this intermediate reacts with C-electrophiles such as CoA-thioesters instead of the proton, the resulting C–C-coupling product 19 can further react either to ketone 17 or after imine isomerisation to amine 21 (Scheme 3, bottom).

**Scheme 4:**
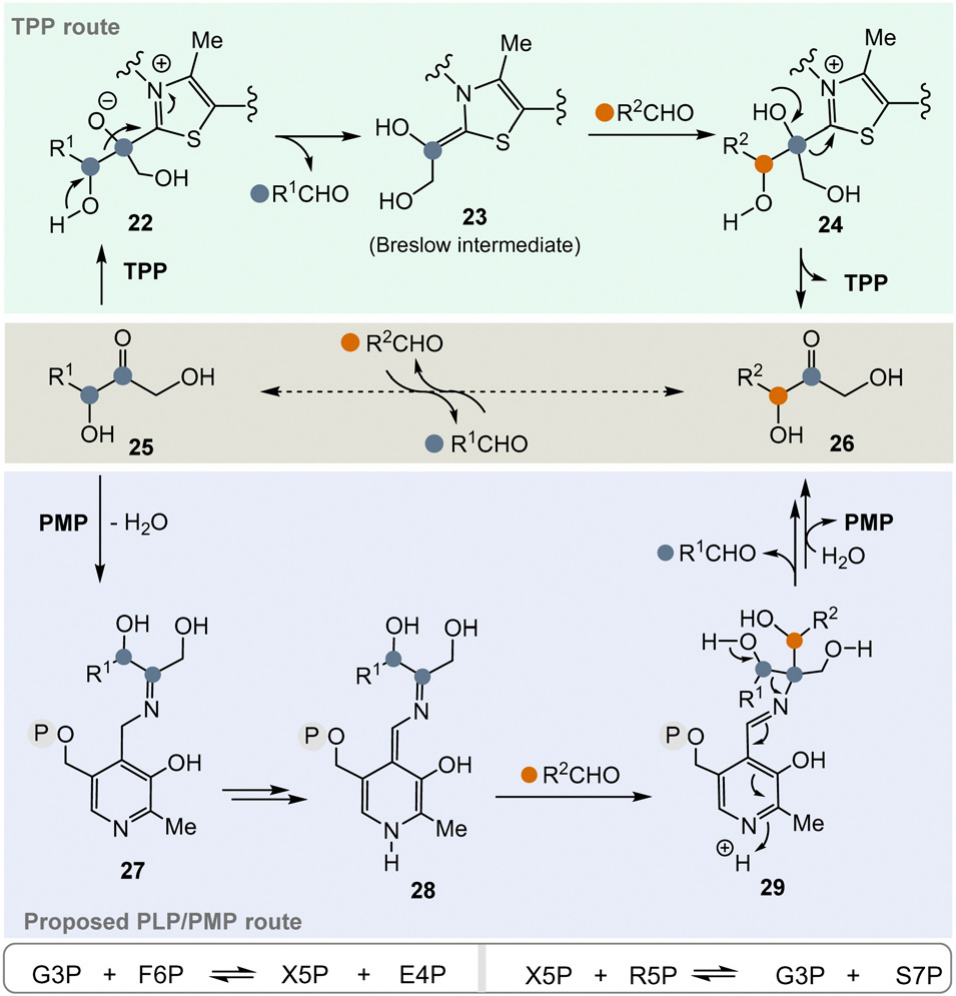
Top: TPP-catalyzed transketolase reaction of hydroxyketone 25 to hydroxyketone 26*via* the Breslow intermediate 23 (resembling 6). Central: Formal acyl exchange (25 → 26). Bottom: Proposed mechanistic route from 25 to 26 that supposedly can be promoted by PMP 2*via* intermediates 27–29 (intermediate 28 formally resembles intermediate 23).

In the pentose phosphate pathway, TPP-dependent transketolases play a key role.^21^ This pathway provides access to pentoses like d-ribose and hydroxyketones 25 take the role of α-ketocarboxylates or α-amino acids described in Scheme 3. In principle, the enzyme reversibly catalyses the exchange of an RCH(OH) group within carbohydrate-based α-hydroxy ketones (25 → 26) (Scheme 4, central), which proceeds *via* “TPP-intermediates” 22–24 (Scheme 4, top). In view of the topic discussed here, a PLP/PMP-dependent mechanism *via* “PLP-intermediates” 27–29 (Scheme 4, bottom) can also be proposed that reflects the one of transketolases. Probably, the most important transketolase reactions promoted by TPP are the reversible transformations of G3P 8 and fructose-6-phosphate (F6P) to xylulose-5-phosphate (X5P) and erythrose-4-phosphate (E4P) as well as those of X5P and R5P 9 to G3P 8 and sedoheptulose-7-phosphate (S7P). According to the hypothesis, the proposed PLP/PMP-dependent mechanism could promote these transformations too.

**Scheme 5:**
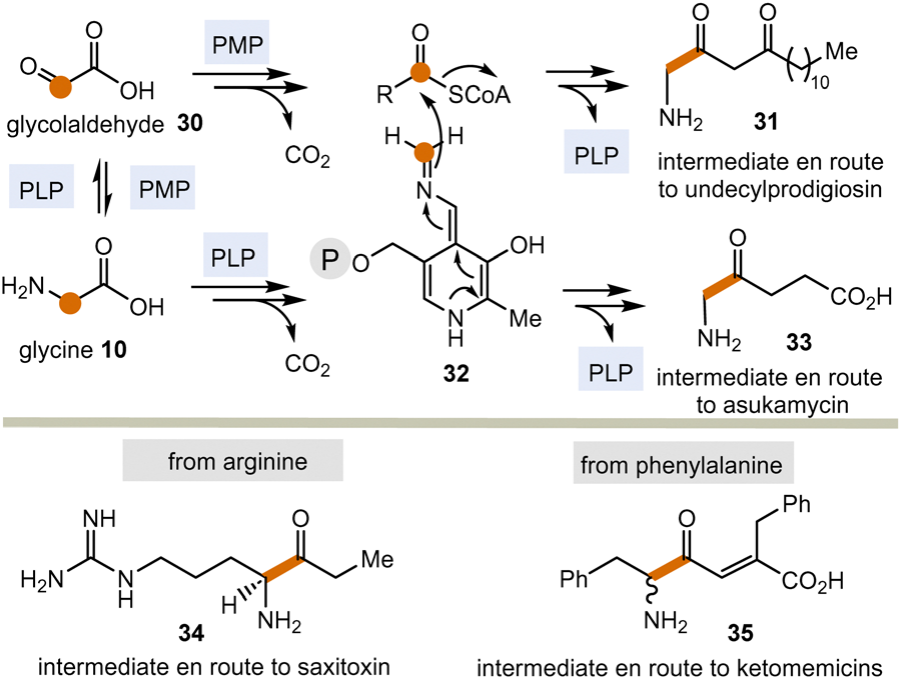
PLP/PMP-promoted biotransformations with formal acyl anion transfer in secondary metabolism (according to Scheme 3; P = phosphate residue).

Today, these kinds of biotransformations are only found in the biosynthesis of secondary metabolites (Scheme 5). Here, amino acids or their transamination products, the corresponding α-ketocarboxylates like glyoxylic acid 30^22^ are the starting points and the C–C bond forming processes lead to products 31 and 33–35*via* intermediate 32 (in analogy to intermediate 18 in Scheme 3). From there, the final natural products undecylprodigiosin,^23^ asukamycin,^24^ saxitoxin^25^ and ketomemicins^26^ are generated.

The biosyntheses of these natural products indicate a potentially broader role of the PLP/PMP 1/2 pair in the past and may thus be regarded vestiges of a mechanistic process that has survived since the emergence of the coenzyme TPP 3.^27^

## 4. Consequences for a possible TPP-free primordial metabolism: a “Gedanken-experiment”

### 4.1. Biosynthesis of amino acids

Having unfolded the mechanistic analogies between TPP-mediated acyl anion chemistry combined with the possibility that the PLP/PMP pair can in principle catalyse similar transformations as “buried” in some biosynthetic pathways of secondary metabolites, it is reasonable to speculate that the biosynthesis of basic building blocks of primary metabolism may have also relied on the PLP/PMP 1/2 pair in some form of primordial metabolism instead of TPP 3.

Among the biosynthetic routes to α-amino acids, the branched representatives valine, leucine and isoleucine but also the aromatic amino acids stand out in that they all require TPP 3.^4,28^ Likewise, the pentose pathway also relies on TPP-dependent transketolases.

#### (a) Branched aliphatic α-amino acids

**Scheme 6:**
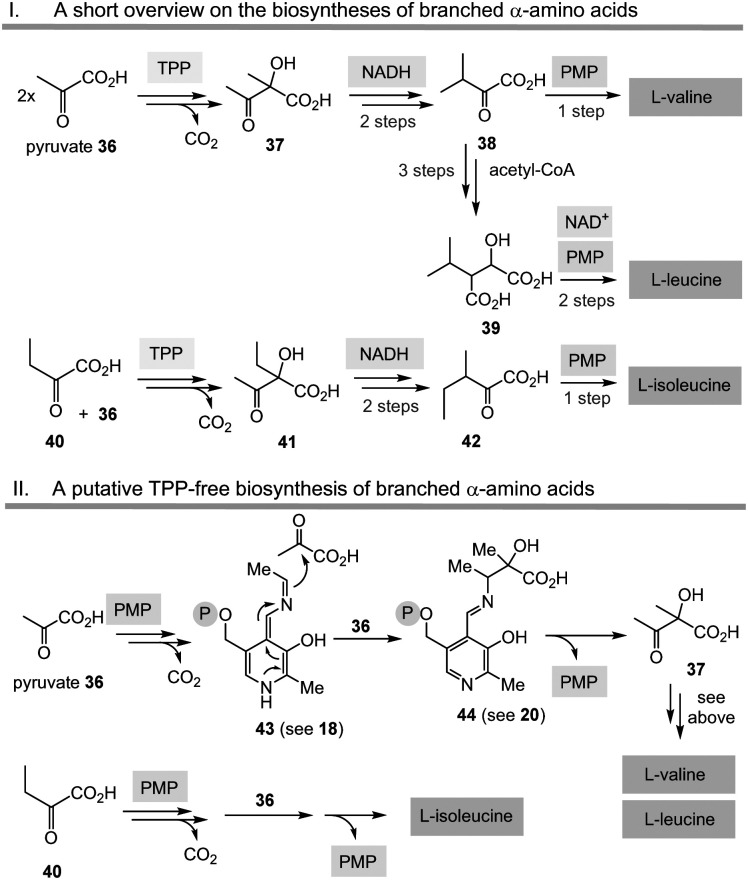
The “Gedanken experiment” conducted for the PLP/PMP-based biosyntheses of valine, leucine and isoleucine (II) and comparison with TPP-dependent routes (I).

In Scheme 6, the biosyntheses of the aliphatic branched α-amino acids l-valine, l-leucine and l-isoleucine are briefly depicted, both for the natural TPP-based route (36 → 37 → 38/39 and 40 → 41 → 42, with mechanistic reference to Scheme 3, top), as well as for the hypothetic PLP/PMP-mediated variants (with 36 → 43 → 44, with mechanistic reference to Scheme 3, bottom). In essence, the carbon skeleton of l-valine is derived from two eq. of pyruvate 36 (with loss of one CO_2_), while l-leucine has recruited its carbon atoms from 2 eq. of pyruvate and an activated acetic acid (with loss of two CO_2_). The biosynthesis of isoleucine relies on pyruvate 36 and α-ketobutyric acid 40.

#### (b) Aromatic amino acids and the shikimate pathway

**Scheme 7:**
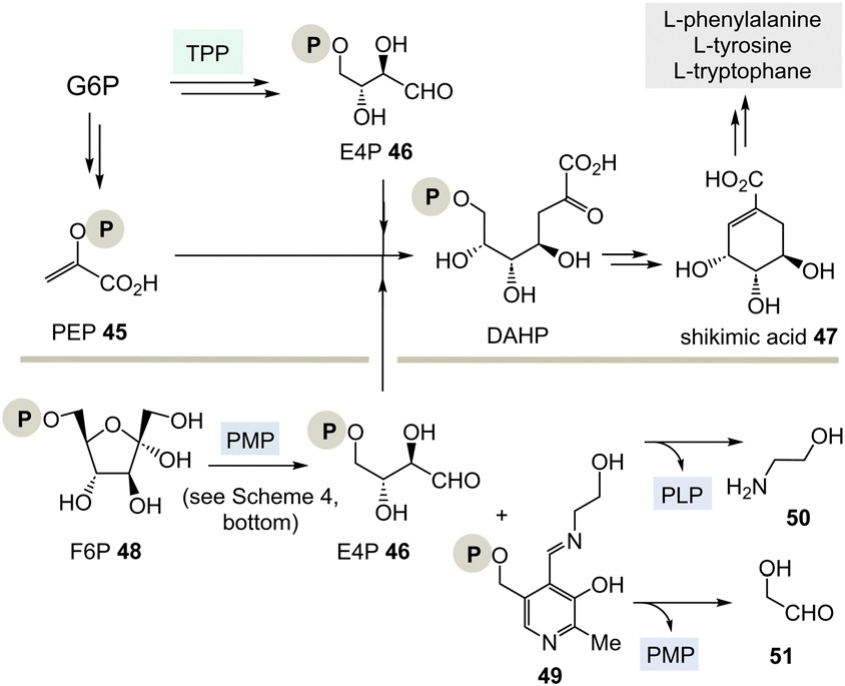
Top: Biosynthesis of shikimic acid 47 from glucose-6-phosphate (G6P) *via* E4P 46 and the role of TPP 3. Bottom: A hypothetical PLP-dependent synthesis of E4P 46 and possible by-products 50 and 51.

**Scheme 8:**
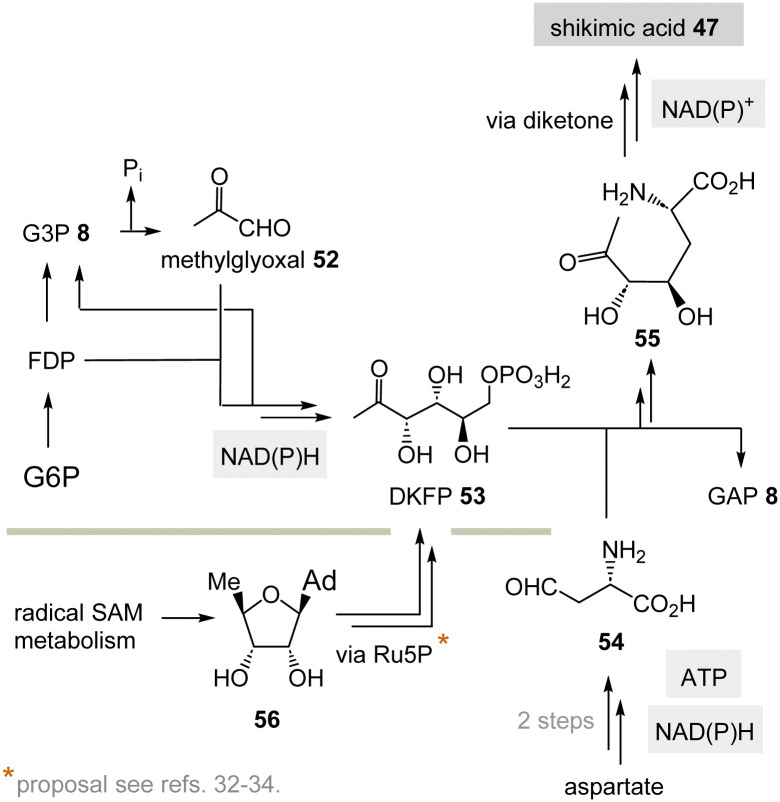
TPP-free biosynthesis of shikimic acid 47 found in some archaea such as *Methanocaldococcus jannaschii* (Ad = adenine).

The aromatic amino acids phenylalanine, tyrosine and tryptophane are formed from shikimic acid 47^8^ which recruits its seven carbon atoms from the transketolase product E4P 46 (see Scheme 4) and phosphoenolpyruvate (PEP, 45) *via* 3-deoxy-d-*arabino*-heptulosonic acid 7-phosphate (DAHP) (Scheme 7, top).^29^ Several TPP-free options to form shikimate 47 are known or can be considered, respectively.

(I) According to Scheme 4, E4P 46 may also be formed by the PLP/PMP couple starting from fructose-6-phosphate (F6P, 48) and would feed through this route into the shikimate pathway. The by-product, *e.g.* one of two tautomeric PMP adducts 49, would hydrolyse to ethanol amine 50 and the other tautomer to glycolaldehyde 51. Potentially, intermediate 49 could also react further with C-electrophiles similar to ketolases.

(II) Some archaea such as *Methanocaldococcus jannaschii* rely on a TPP-free biosynthesis to shikimic acid 47.^30^ 6-Deoxy-5-ketofructose-1-phosphate 53 is a key intermediate that is formed from G3P 8 and fructose-1,6-diphosphate (FDP)^31^ (Scheme 8). Phosphate elimination in G3P 8 furnishes methylglyoxal 52 and a transaldolase reaction provides 6-deoxy-5-ketofructose-1-phosphate (DKFP, 53) and G3P 8. A second aldolase-promoted coupling of DKFP 53 with l-aspartate semialdehyde 54 yields α-amino acid 55. From there, several steps including a cyclisation analogous to the common pathway furnish shikimic acid 47.^8^

Recently, White and coworkers suggested that in the methanogenic archaeon *M. jannaschii* 5-deoxyadenosine 56 could well serve as a precursor for the biosynthesis of aromatic amino acids (5dAdo salvage).^32–34^ They based their proposal on the observation that the genes encoding for DAHP synthase and for the canonical pentose phosphate pathway are absent.

Consequently, E4P 46 cannot serve as a precursor for the biosynthesis of aromatic amino acids.^35^ However, radical *S*-adenosylmethionine (SAM) enzymes make up almost 2% of the *M. jannaschii* genome. Typically, 5′-deoxyadenosine 56, an inhibitor of radical SAM enzymes, is the major by-product of this biochemistry. The group hypothesised that starting from 56 an alternative biosynthetic pathway to aromatic amino acids *via* deoxyhexoses in *M. jannaschii* is conceivable.^34^

### 4.2. The pentose phosphate pathway

**Scheme 9:**
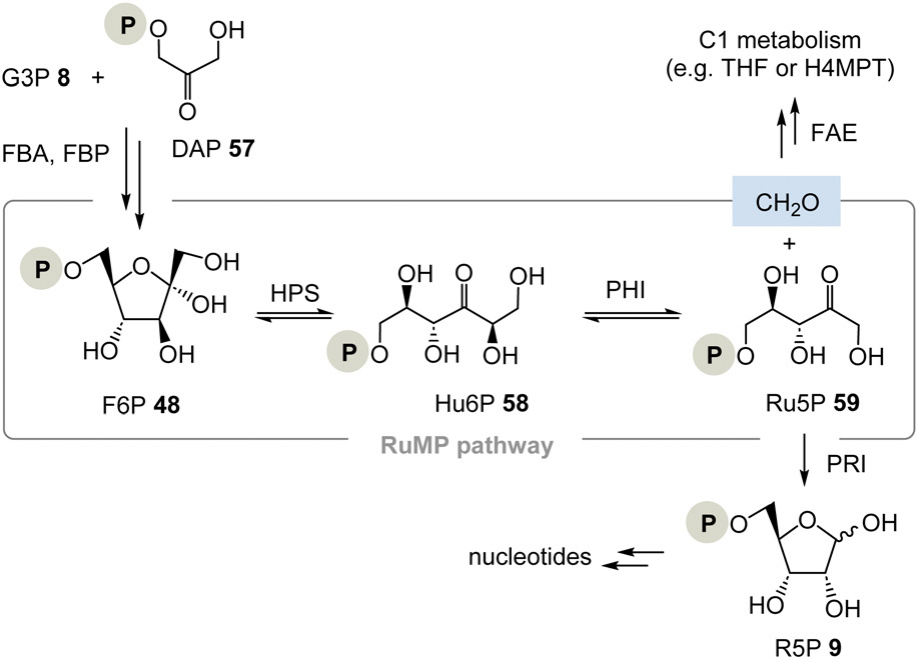
The ribulose monophosphate (RuMP) pathway provides ribose-5-phosphate 9 and does not require TPP 3 (FBA = fructose-1,6-bisphosphate aldolase, FBP = fructose-1,6-bisphosphatase, FAE = formaldehyde activating enzyme, PRI = ribose-5-phosphate isomerase, H4MPT = tetrahydromethanopterin).

The pentose phosphate pathway is a second key element of primary metabolism and in its reversible part monosaccharides of different chain lengths are transformed into each other by the TPP-dependent transketolase biotransformation (examples are found in Scheme 4, lowermost bottom).^36^ This pathway provides access to pentoses like d-ribose and two scenarios for a TPP-free carbohydrate metabolism can be considered.

(I) As discussed above and depicted in Scheme 4, a PLP/PMP-dependent mechanism can also be proposed that reflects the one of transketolases.

(II) The ribulose monophosphate (RuMP) pathway is an alternative biosynthetic route to pentoses and it has created only little attention to date (Scheme 9).^37^ For example, in the archaeon *Thermococcus kodakarensis*, the pentose phosphate pathway is missing. This analysis, however, does not conclude that their metabolism is necessarily TPP-free as was analysed for *Borrelia* and *Rickettsia*.^37*b*^

The RuMP is based on the aldol coupling of ribulose-5-phosphate (Ru5P, 59) with formaldehyde to furnish 3-hexulose-6-phosphate (Hu6P, 58). This step is catalysed by 3-hexulose-6-phosphate synthase (HPS). Hu6P 58 is then converted to F6P 48 by 3-hexulose-6-phosphate isomerase (PHI). It is recognised as a prokaryotic pathway for formaldehyde fixation and detoxification. HPS and PHI homologs are also found in a variety of archaeal strains, and recent studies provided evidence that the reverse reaction of formaldehyde fixation, *i.e.*, Ru5P 59 synthesis from fructose 6-phosphate 48, may function in some archaeal strains whose pentose phosphate pathways are imperfect.^38^

### 4.3. A TPP-free primary metabolism – a broader view

The TCA cycle (Krebs cycle) is a common reservoir of building blocks serving the biosynthesis of amino acids. In addition, the pentose phosphate pathway is also central to the primary metabolism and this relies (in part) on the two coenzymes PLP/PMP 1/2 and TPP 3.

**Scheme 10:**
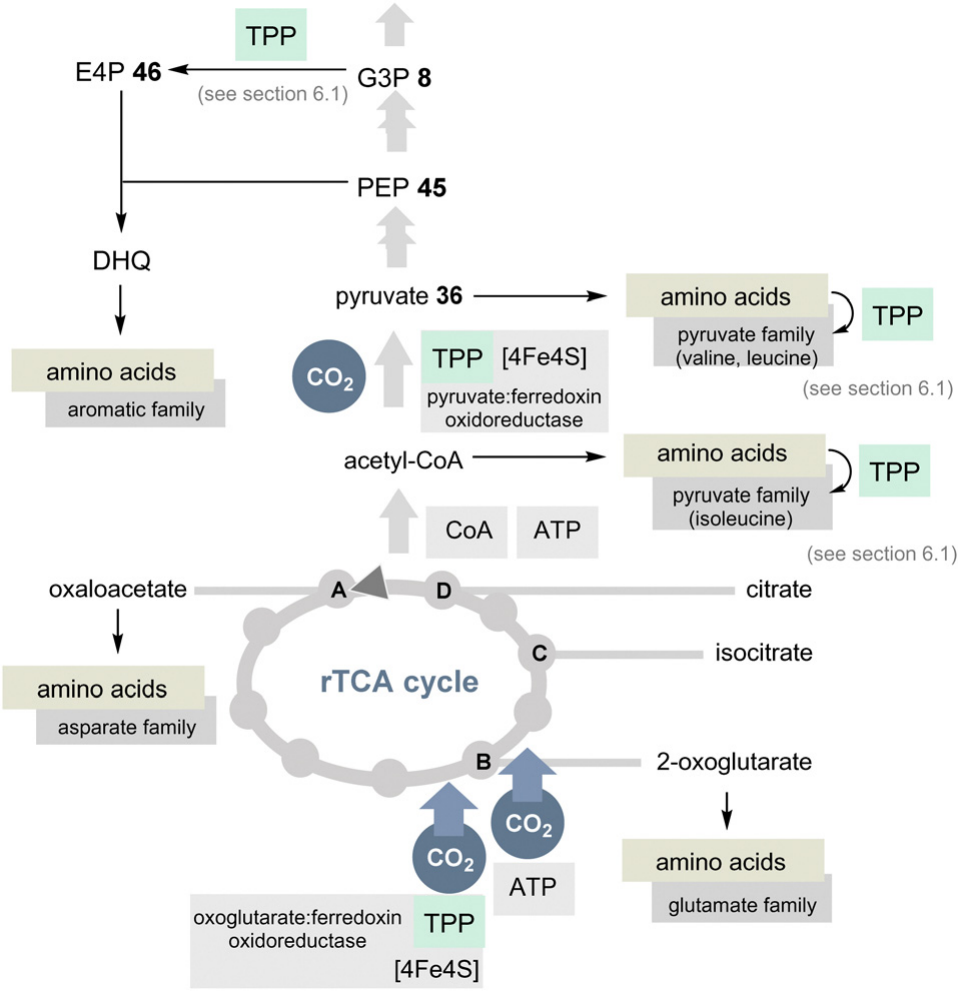
Overview of the rTCA cycle with a link to the 20 encoded amino acids (except histidine). The role of TPP is highlighted in light green (DHQ = dehydroquinate).

The TCA cycle utilises carbohydrates and oxidises them to CO_2_ and water. Noteworthily, amino acids are also degraded and the biosynthesis of glutamate from glucose occurs in the usual TCA cycle too. In contrast, the rTCA cycle picks up CO_2_ and water to form biomolecules (Scheme 10).^39,40^ The rTCA cycle is found in bacteria and archaea and because of its anabolic character it has been called a blueprint for a primordial form of C1 fixation.^41^

In view of the present account, two enzymes need to be highlighted to distinguish the rTCA cycle from the TCA cycle. 2-Oxoglutarate:ferredoxin oxidoreductase is a TPP-dependent enzyme and an additional iron–sulfur [4Fe–4S] cluster that transfers CO_2_ to succinyl CoA to furnish 2-oxoglutarate and coenzyme A (Scheme 10).^42^ The other step of CO_2_ fixation within the rTCA cycle is promoted by 2-oxoglutarate carboxylase. It first activates bicarbonate as a mixed anhydride with phosphate and, after transfer to biotin, C3 in 2-ketoglutarate is carboxylated to furnish oxalosuccinate. This chemically unstable species is then reductively converted into isocitrate by the coenzyme NADH.

A second TPP-dependent CO_2_ fixing enzyme is pyruvate:ferredoxin oxidoreductase (PFOR) found in bacteria and archaea that in addition requires three [4Fe–4S] clusters as well as coenzyme A. It reversibly converts pyruvate into acetyl-CoA and carbon dioxide (Scheme 10).^40*b*,43^

If TPP 3 emerged at a later stage of evolution, it seems doubtful that the rTCA cycle could have played a primordial role in C1 fixation. Furthermore, this would have a direct impact on the biosynthesis of most proteinogenic amino acids, especially those that require TPP 3. The adapted coenzyme requirement for the modified biosyntheses of the branched aliphatic and aromatic α-amino acids was discussed above and will not be considered further here.^44^

**Scheme 11:**
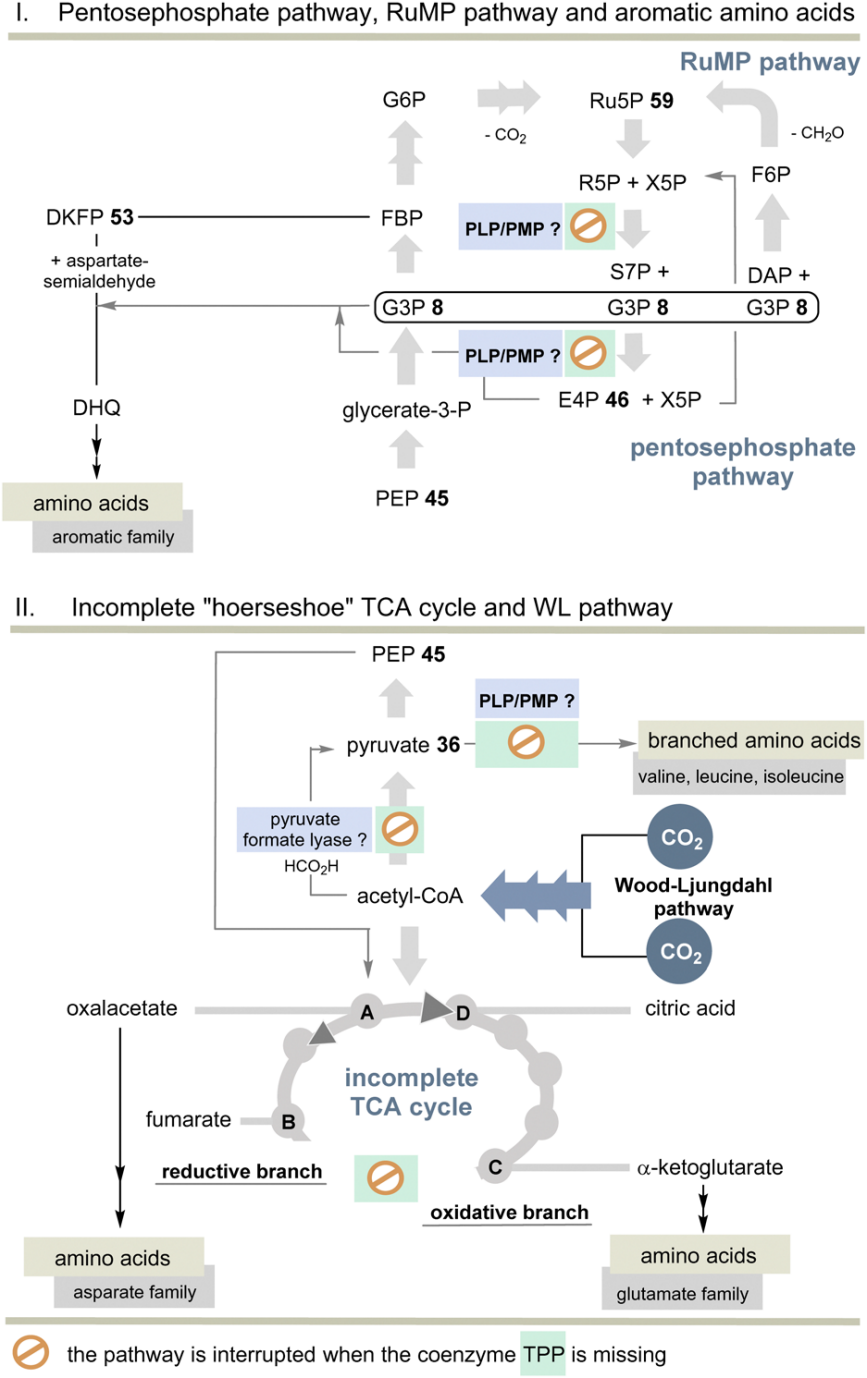
Top: Overview of pentose phosphate and RuMP pathways and the biosynthesis of aromatic amino acids without TPP. Bottom: Overview of the incomplete “horseshoe” TCA cycle and the link with the WL pathway. The steps that do not require TPP compared to the TCA and rTCA cycles are specifically marked (see also Scheme 10).

But the non-cyclic putative evolutionary precursor of the TCA cycle, also known as incomplete “horseshoe” TCA, could help solve the TPP problem. It was found, *e.g.*, in the strictly anaerobic bacterium *Elusimicrobium minutum*^45^ (Scheme 11). Here, the link between 2-oxoglutarate and fumarate is interrupted by the absence of α-oxoglutarate dehydrogenase and succinate dehydrogenase, as well as the intermediates succinyl-CoA and succinate so that an oxidative half-cycle to α-oxoglutarate and a second reductive half-cycle to fumarate remain intact. The reductive branch of the incomplete TCA cycle is initiated by the interconversion of oxaloacetate, malate, and fumarate. Consequently, TPP 3 is not required and this metabolic branching that was found in many anaerobes is primarily used for the biosynthesis of various amino acids, but not necessarily for all proteinogenic ones.

Indeed, *E. minutum*^45^ is able to form glutamate, glutamine and proline (glutamate family), aspartate, cysteine and threonine (aspartate family), glycine, alanine and serine (pyruvate family), as well as lysine and histidine by being fed through the incomplete TCA cycle. In addition, the organism depends on the external influx of arginine, asparagine and methionine, as well as the aromatic and alkyl-branched amino acids. Here we return to the topic of this account as the biosynthesis of these latter amino acids depends on TPP 3.

The other topic relates to pyruvate:ferredoxin oxidoreductase that also depends on TPP 3. Are there other ancient pathways in nature to generate acetyl-CoA? A second important candidate for C1 fixation in the early evolution of life is the linear Wood–Ljungdahl pathway (WL-pathway) which takes two equivalents of CO_2_ and reductively generates acetyl-CoA. It is composed of two branches, namely, the methyl branch and the CO branch (carbonyl branch),^46^ and it has been associated with LUCA^3^ and is considered a blueprint for a primordial form of C1 fixation.^47^ This metabolic pathway consists of two branches in which CO_2_ is first reductively converted to formate (methyl branch) or to CO (carbonyl branch).^48^ In the former, CO_2_ reduction is brought about by the pterin-based molybdenum cofactor.^49^ Several steps of this linear C1 fixation pathway rely on metal-containing cofactors based on Fe, Ni, Co and Mo.^50^ Next, we need to take a look at pyruvate formate lyase (PFL)^51^ which is known for the TPP-independent transformation of pyruvate 36 to acetyl CoA and is thus a substitute for PFOR (Scheme 11). It was recently found that the PFL reaction can also take place *in vivo* in the reversible mode.^52^ Acetyl-CoA from the WL pathway could thus flow into the carbohydrate metabolism.

## 5. Conclusions

As outlined here, the coenzyme pair PLP/PMP 1/2, supposedly biosynthetically more ancient than the coenzyme TPP 3, could have acted as an N-heterocyclic carbene analogue in times when early forms of metabolism could not have relied on TPP. The current biological role of PLP/PMP 1/2 is mainly dedicated to the field of amino acids and biogenic amines. Still examples can be found today, especially in the field of secondary metabolites, in which PLP/PMP-mediated biotransformations proceed *via* Breslow-analogous intermediates. In essence, various facts and observations indicate a possible chemical and evolutionary predecessor role of the PLP/PMP 1/2 pair for TPP 3.

But what could have been a driving force for evolution to progress in the direction of TPP? According to the mechanisms depicted in Schemes 3 and 4, the PLP/PMP alternative reveals a great degree of ambiguities that lead to a larger number of different possible routes due to the imine tautomerism of the PLP/PMP adducts. Each of these intermediates is prone to hydrolysis under aqueous conditions. Was there a quest for a more “robust” acyl anion methodology? TPP-catalysed biotransformations proceed through clearly defined reaction pathways *via* the Breslow intermediate 5. But PLP/PMP could have played a very important role in times of a simple primordial metabolism, and this would also include acyl anion transfer chemistry, but due to these mechanistic considerations this role was likely carried out very inefficiently.

Interestingly, recent developments in organocatalysis have discovered the PLP/PMP pair to serve as an interesting lead structure for promoting catalysis along the lines of the coenzyme (see Fig. 1). This also included C–C coupling chemistry displayed in Scheme 5. However, these developments have focussed on organic synthesis and are thus typically performed in organic solvents.^53^

The considerations outlined here are speculative in every respect, as they attempt to take a glimpse at a metabolism before the appearance of LUCA.^3^ Suggestions about a possible primordial metabolism arising from the approach taken here, which is based on chemical considerations related to metabolism, are of course speculative and tend to be provocative, as are also other approaches, including phylogenetic analyses. The present report can do no more than provide new ideas on primordial forms of life irrespective of whether they are right or wrong. Albert Eschenmoser would have exclaimed: “*The origin of Life cannot be discovered, it has to be reinvented*.”^54^

## Conflicts of interest

There are no conflicts to declare.

## Supplementary Material
